# Caspase-1-Like Regulation of the proPO-System and Role of ppA and Caspase-1-Like Cleaved Peptides from proPO in Innate Immunity

**DOI:** 10.1371/journal.ppat.1004059

**Published:** 2014-04-10

**Authors:** Miti Jearaphunt, Chadanat Noonin, Pikul Jiravanichpaisal, Seiko Nakamura, Anchalee Tassanakajon, Irene Söderhäll, Kenneth Söderhäll

**Affiliations:** 1 Department of Comparative Physiology, Uppsala University, Uppsala, Sweden; 2 Aquatic Molecular Genetics and Biotechnology Laboratory, National Center for Genetic Engineering and Biotechnology (BIOTEC), National Science and Technology Development Agency, Pathumthani, Bangkok, Thailand; 3 Center of Excellence for Molecular Biology and Genomics of Shrimp, Department of Biochemistry, Faculty of Science, Chulalongkorn University, Bangkok, Thailand; 4 Science for Life Laboratory, Department of Comparative Physiology, Uppsala University, Uppsala, Sweden; Stanford University, United States of America

## Abstract

Invertebrates rely on innate immunity to respond to the entry of foreign microorganisms. One of the important innate immune responses in arthropods is the activation of prophenoloxidase (proPO) by a proteolytic cascade finalized by the proPO-activating enzyme (ppA), which leads to melanization and the elimination of pathogens. Proteolytic cascades play a crucial role in innate immune reactions because they can be triggered more quickly than immune responses that require altered gene expression. Caspases are intracellular proteases involved in tightly regulated limited proteolysis of downstream processes and are also involved in inflammatory responses to infections for example by activation of interleukin 1ß. Here we show for the first time a link between caspase cleavage of proPO and release of this protein and the biological function of these fragments in response to bacterial infection in crayfish. Different fragments from the cleavage of proPO were studied to determine their roles in bacterial clearance and antimicrobial activity. These fragments include proPO-ppA, the N-terminal part of proPO cleaved by ppA, and proPO-casp1 and proPO-casp2, the fragments from the N-terminus after cleavage by caspase-1. The recombinant proteins corresponding to all three of these peptide fragments exhibited bacterial clearance activity *in vivo*, and proPO-ppA had antimicrobial activity, as evidenced by a drastic decrease in the number of *Escherichia coli* in vitro. The bacteria incubated with the proPO-ppA fragment were agglutinated and their cell morphology was altered. Our findings show an evolutionary conserved role for caspase cleavage in inflammation, and for the first time show a link between caspase induced inflammation and melanization. Further we give a more detailed understanding of how the proPO system is regulated in time and place and a role for the peptide generated by activation of proPO as well as for the peptides resulting from Caspase 1 proteolysis.

## Author Summary

Melanization is an important reaction in most multicellular organisms, both animals and plants. The initiation steps of this reaction in invertebrates are catalyzed by the prophenoloxidase (proPO) activating system a proteolytic enzyme cascade, which primary function is to recognize cell wall products from microorganisms and respond by activation of the system and generation of immune effector molecules. This cascade requires careful regulation to achieve spatial and temporal control to avoid dangerous side effects. We here show that a Caspase1-like enzyme can inactivate proPO when ppA is not activating the proPO to avoid deleterious effects and further we show for the first time that the N-terminal peptide from ppA cleavage of proPO (activation of proPO) has an important biological function as also the Caspase1 cleaved fragments. Our results also show that Caspase 1-induced inflammatory response is evolutionarily conserved and is linked to melanization.

## Introduction

Melanization is the result of the oxidation of mono- and/or diphenols by a redox enzyme, often phenoloxidase, and it is an important reaction in most multicellular organisms, both animals and plants. Intruding microorganisms are frequently melanized in invertebrates, and during this process, low-molecular-weight phenolic substances are converted into polymeric melanin in a multi-step chain of reactions. The initiation steps of this reaction are catalyzed by the prophenoloxidase activating system (proPO system), and other steps occur spontaneously. The proPO system is a proteolytic enzyme cascade and its primary function is to recognize minuscule amounts (picograms per liter) of cell wall products from microorganisms (lipopolysaccharide (LPS), peptidoglycan (PGN) and glucans) and respond to the microorganism by activation of the system and the subsequent generation of immune factors. This cascade requires careful regulation to achieve spatial and temporal control to avoid dangerous side effects [1]. A number of regulatory factors are involved in controlling the activation of the proPO system, including proteinase inhibitors [2] and the melanization-inhibiting protein (in insects, crayfish and shrimp) [3], [4], [5].

The importance of melanization (proPO system) in controlling a number of specific host–pathogen encounters has been demonstrated over the past few years. One example of this is the bracovirus protein Egf1.0, which inhibits the prophenoloxidase (proPO)-activating proteinase in the insect *Manduca sexta* [6]. Two other recent examples are found in the parasitoid wasp *Leptopilina boulardi*, which targets the *Drosophila* phenoloxidase cascade by producing a specific serpin inhibitor [7], and in the bacterium *Photorhabdus luminescens*, which secretes a small organic molecule that acts as a negative regulator of PO activity [8]. In addition, a pathogenic *Aeromonas hydrophila* strain becomes highly virulent to crayfish when the PO transcript levels are experimentally reduced [9]. The proPO activation system, or melanization cascade, bears functional resemblance to the complement system, although the final reaction, melanization, is different. Intriguingly, recently we succeeded in showing that all of the steps in the proPO-cascade in *Tenebrio molitor* are shared with the proteinase cascade that leads to the activation of the Toll pathway for the production of antimicrobial peptides [10]. This shared cascade has been confirmed in several other insects [11], [12]. In the present study, we found that caspases are very important for the rapid degradation of proPO, which prevents oxidation in places where it is not appropriate.

Caspases, or cysteine-aspartic proteases, are a family of the cysteine proteases that are known for their function in apoptosis [13]. Some caspases are involved in the inflammatory system via the regulation of pro-inflammatory cytokines, and these caspases are necessary regulators of the unconventional secretion of leaderless proteins [14], [15]. In humans, caspase-1 is not only required for the activation of pro-interleukin (IL)-1β and pro-IL-18, but also functions as an activator of nuclear factor of the kappa-enhancer in B-cells (NF-κB) and p38 mitogen-activated protein kinase (MAPK) [16]. Interleukin-1β is produced as a cytosolic precursor and is dependent on caspase-1 cleavage for its activation and secretion [15], [17]. The proPO is also produced as a leaderless protein, most likely in the cytosol, and is secreted by an unknown mechanism. We, therefore, searched the sequence for caspase-1 cleavage sites and found two in the middle of the Cu-binding region. Therefore, we asked whether caspase-1-like cleavage of proPO is involved in the regulation of PO activity. We also asked whether the caspase-cleaved fragments have biological functions and whether these fragments are involved in immune functions even in the absence of PO activity. We also studied whether the peptide fragments generated by the cleavage of proPO into active PO by the prophenoloxidase activating enzyme (ppA), which gives a peptide of approximately 20 kD, might have some immune function during or immediately prior to melanization.

Our studies provide new information about the function of caspase-1-like activity in freshwater crayfish, in which it acts as a negative regulator of the proPO system. For the first time, we provide results showing that the fragments resulting from caspase or ppA cleavage have important biological functions.

## Results

### Localization of proPO and detection of caspase-1-like protein in crayfish

**Figure 1:**
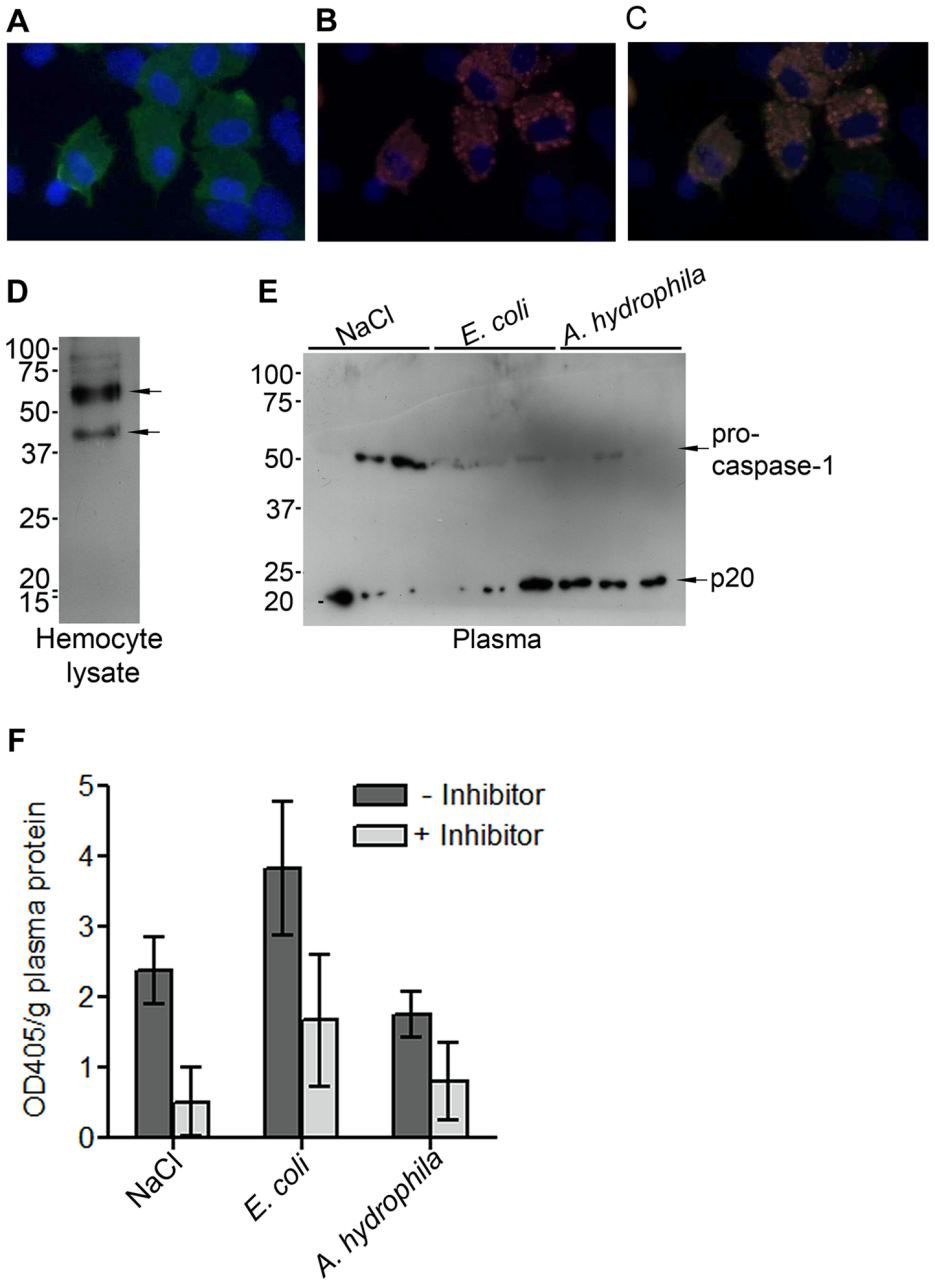
Expression of proPO and caspase-1-like protein in crayfish *P. leniusculus*. ProPO is highly expressed in the cytoplasm of some granular cells, as shown in green in (A). Counterstaining for granules (B), kazal proteinase inhibitor (red) and nuclear staining of the cells, DAPI, (blue) (C). Western blot showing the presence of procaspase-1-like proteins (40 and 50 kDa) in the hemocytes of untreated crayfish (D). The 50 kDa band was detected in crayfish plasma and the level of this band was decreased in bacteria injected crayfish (E). In addition, the active subunit of caspase, p20, was detected in plasma (E). Plasma caspase-1 activity was determined, and the activity was reduced by the addition of the caspase-1 inhibitor, Z-YVAD-FMK (F). The values shown are mean of five samples and the bars indicate SEM. The statistical analysis was performed using one-way ANOVA.

ProPO, the inactive form of PO, is present in crayfish hemocytes, especially in the granular cells (GC). GCs are densely filled with granules, as indicated by their name. Upon activation by different environmental challenges such as microbes, exocytosis is induced, which causes the release of several proteins from the granules of the GCs and the release of proPO into the external milieu [18]. Immunostaining of proPO in GCs revealed that proPO is present in the cytoplasm but not in the granules, and not all GC cells express proPO (Figures 1A–C). ProPO is cleaved extracellularly to produce active PO upon activation by ppA. However, the mechanism by which proPO is released is still unknown. In beet armyworm and *Drosophila*, prostaglandins and JNK can stimulate cell lysis and subsequent proPO release [19], [20]. In mammal, there are many reports showing that inflammasomes and caspase-1 activation are involved in the secretion of proteins without signal peptides [21], [22]. Thus, we asked whether caspase-1 plays a role in proPO release and/or regulation. To answer this question, the presence of caspase-1 in the crayfish was examined. The transcriptome analysis of the freshwater crayfish *P. leniusculus* (unpublished data) revealed the presence of a translated amino acid sequence that has 36% identity to *Drosophila* caspase interleukin-1 beta converting enzyme (GenBank: NP524551). Additionally, by using an antibody against human caspase-1, two bands with sizes about 40 and 50 kDa were detected from crayfish hemocyte lysate (Figure 1D). These bands are probably two isoforms of the crayfish procaspase-1 like proteins. In comparison, in human six different isoforms of caspase-1 have been found. The 50 kDa procaspase-1 like protein was also detected in crayfish plasma and the level of this protein in plasma was decreased 1 h after an injection of *E. coli* or *A. hydrophila* compared to the control (0.15 M NaCl) (Figure 1E). Notably, when the 50 kDa band decreased, a 20 kDa band appeared in the plasma (Figure 1E). The 20 kDa band is similar in size to the p20 subunit of mammalian caspase-1, which is the active subunit of this protein. The active caspase-1 could only be detected in the supernatant and not in human keratinocyte lysates [15]. This is probably because it is rapidly degraded or released to the outside of cells and is therefore not present in cell lysates. Another explanation may be that active caspase-1 has a very short half-life, and therefore, its concentration under physiological conditions is very low [22], [23]. Caspase-1 activity was also found to be slightly increased in plasma at 1 h after injection with *E.coli* but no statistically significant difference could be observed (Figure 1F) and this activity could be decreased by incubation of the caspase-1 inhibitor, Z-YVAD-FMK.

### Caspase-1 cleavage site of crayfish proPO

**Figure 2:**
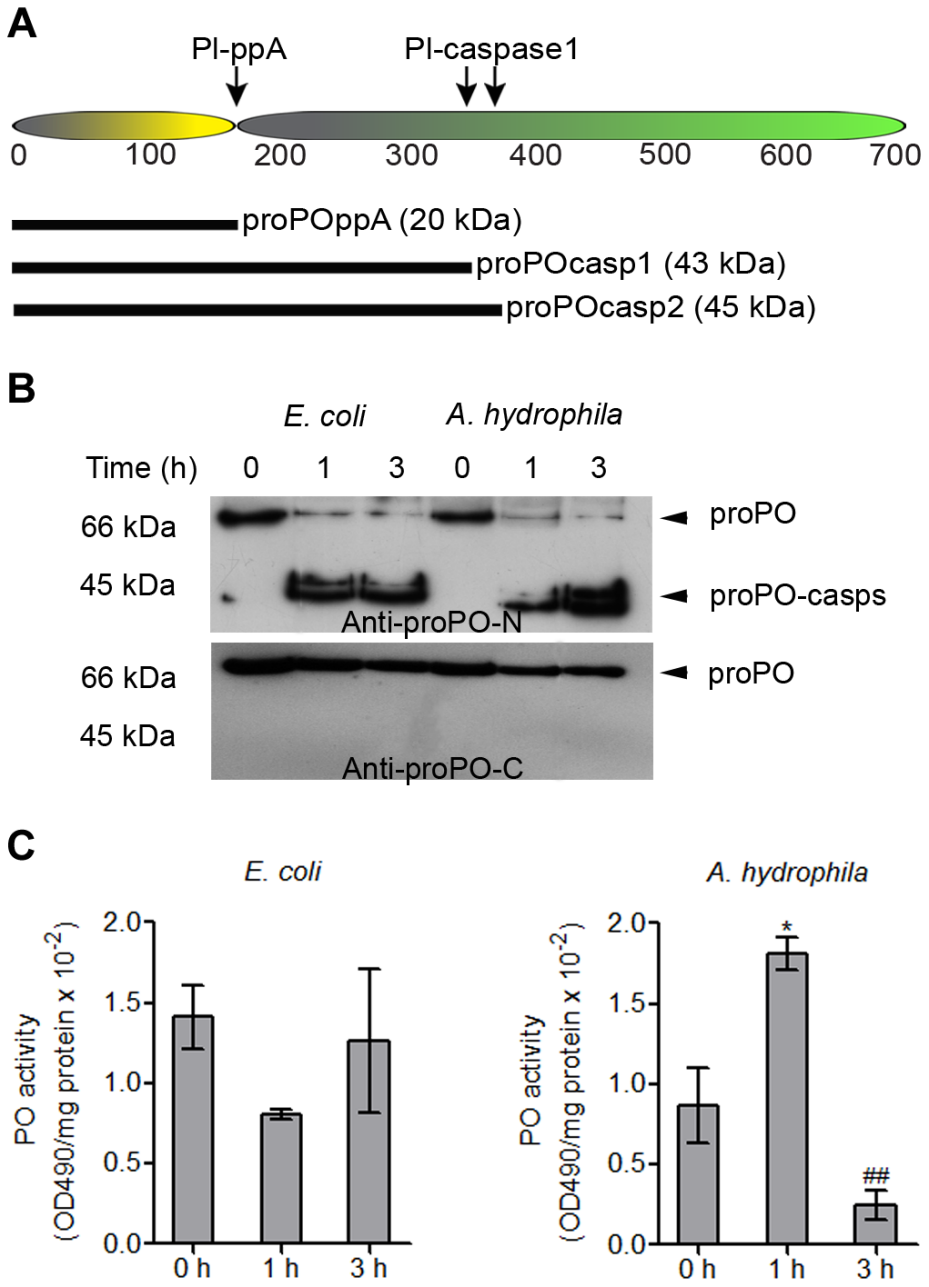
Cleavage site of caspase-1-like protein and the presence of proPO-casp fragments in plasma and the plasma PO activity after bacterial injection. Schematic figure of the proPO protein indicating the cleavage site for ppA and the putative cleavage sites for a caspase-1-like enzyme (A). Recombinant proPO-casp1 and proPO-casp2 fragments were expressed with a hexahistidine-tag at the N-terminus, and proPO-ppA was expressed with a thioredoxin tag at the N-terminus. Crayfish (N = 5) were injected with *E. coli* or *A. hydrophila* B1. Hemolymph was collected at 0, 1, and 3 h after injection. The proPO level in the plasma decreased over time, whereas the levels of proPO-casps increased (A). There was no significant difference in PO activity in the plasma from *E. coli*-infected crayfish. In contrast, the PO activity significantly increased at 1 h and markedly decreased at 3 h after *A. hydrophila* B1 infection, in correlation with the appearance of proPO-casp fragments (B). The statistical analysis was performed using one-way ANOVA. * *P*<0.05, significant difference compared with the 0 h time point, ^##^ *P*<0.01, significant difference compared with the 3 h time point. The values shown in (C) are mean of five samples and the bars indicate SEM.

Our results suggest that caspase-1-like activity is present in crayfish and that this activity can be activated during infection. As mentioned above, the activation of caspase-1 in vertebrates is involved in the secretion of several proteins, such as IL-1 β, but to date, no such mechanism has been identified in invertebrates, although the secretion of leaderless proteins such as proPO [18] and *Pl*-β-thymosins [24] has been observed. Therefore, the amino acid sequence of proPO was analyzed to determine whether there is a potential caspase-1 cleavage site in the proPO sequence. Two caspase-1 cleavage sites were found, after amino acids 363 and 389, which would give rise to two N-terminal proPO fragments with predicted sizes of approximately 42 kDa (proPO-casp1) and 45 kDa (proPO-casp2), respectively (Figure 2A). These two cleavage sites are located downstream of the cleavage site for ppA and would give rise to a small N-terminal fragment (20 kDa of proPO-ppA) and a C-terminal active PO [25]. Therefore, cleavage as the result of caspase-1-like activity has the potential to reduce the PO activity and act as a negative regulator of the proPO system.

### The presence of proPO-caspase fragments in the plasma and plasma PO activity after bacterial infection *in vivo*

To investigate whether any proPO-caspase fragment (proPO-casp) is released from hemocytes, plasma proteins from bacteria-infected crayfish were subjected to western blotting. The results in Figure 2B show that two caspase-cleaved proPO fragments were present in the plasma after bacterial infection and that the levels of both proPO-casps increased with time, whereas the plasma proPO level decreased. Moreover, the level of proPO-casp1 was higher than that of proPO-casp2 at all time points. This result suggests that proPO-casp2 may be degraded faster than proPO-casp1 *in vivo*. When non-virulent *E. coli* was injected, the released proPO was rapidly cleaved by a caspase, and high levels of the fragments were detected at 1 and 3 hours post injection. In contrast, the injection of a virulent *A. hydrophila* strain resulted in slower caspase cleavage, and a high level was reached after 3 hours (Figure 2B). Interestingly, when the levels of the C-terminal fragments of proPO in the samples were analyzed, neither active PO, nor proPO-casps could be detected, and only inactive proPO was found in the plasma. This result suggests that the C-terminal fragments of proPO produced by caspase-1-like cleavage, as well as ppA cleavage, are degraded rapidly.

Because cleavage by caspase-1 may reduce PO activity, the enzyme activity in the plasma was measured after bacterial infection (Figure 2C). The PO activity decreased after *E. coli* infection, but there was no significant difference, and interestingly, the plasma PO activity was significantly higher at 1 h after *A. hydrophila* infection, at time at which the plasma proPO-casp levels were low, and the enzyme activity markedly decreased by 3 h, when the levels of these fragments were higher. Notably, all animals died 4–6 hours after *A. hydrophila* infection.

### Ca^2+^-dependence of proPO-casps release *in vitro*

**Figure 3:**
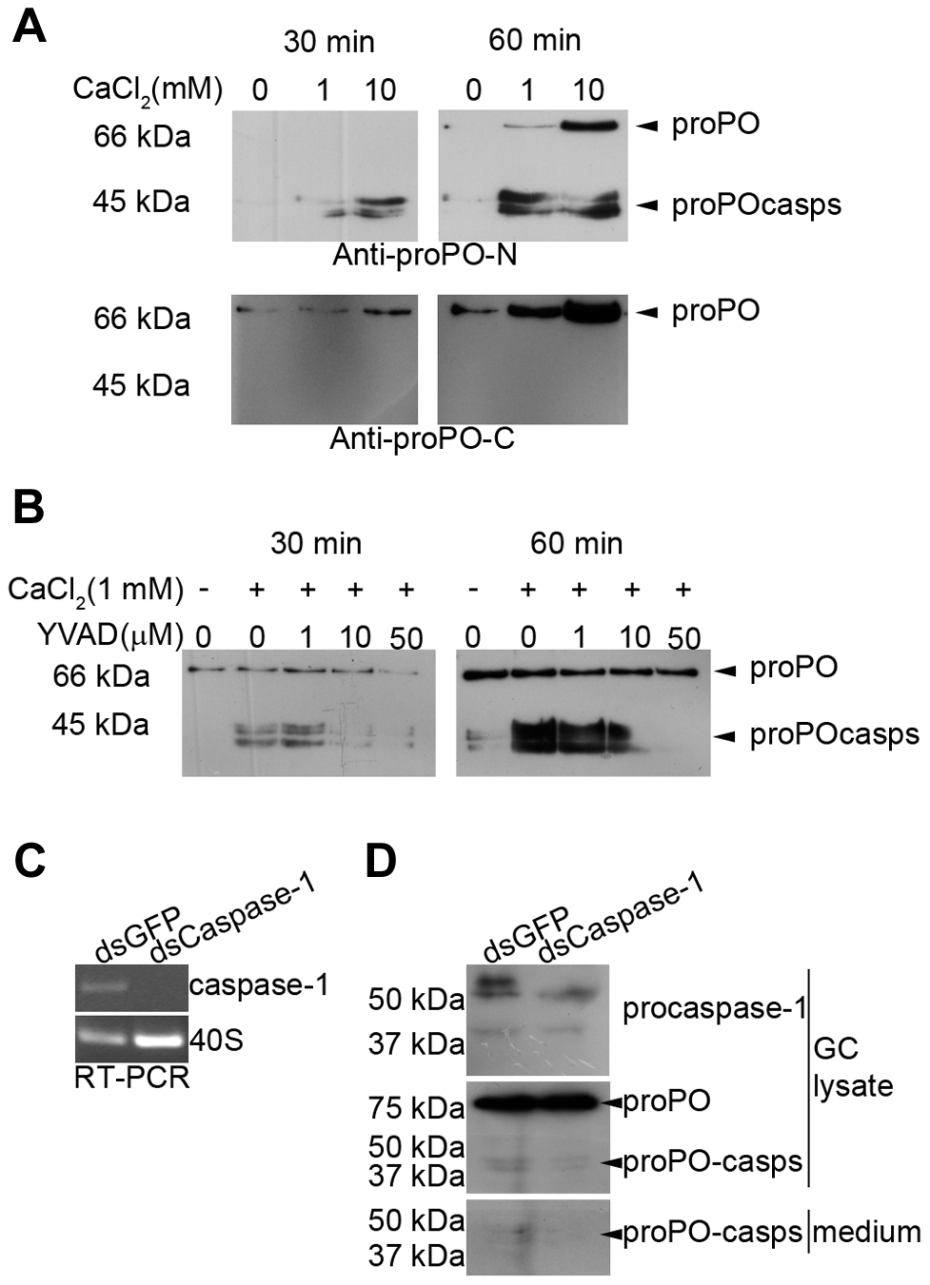
Ca^2+^ dependence of proPO-casp release and the inhibitory effect of a caspase-1 inhibitor and caspase-1 knockdown *in vitro*. Granular cells (GC) were incubated in buffer containing different concentrations of Ca^2+^. The presence of proPO-casps outside the cells was examined in medium at 30 and 60 min. The release of proPO-casps was Ca^2+^ and time dependent (A) and was inhibited when the cells were preincubated for 30 min with the caspase-1 inhibitor Z-YVAD-FMK prior to Ca^2+^ addition (B). The caspase-1-like transcript was completely decreased after 65 h of caspase-1 dsRNA (dsCaspase-1) treatment when compared to the dsGFP control (C). The level of proPO-casp fragments were decreased in both cell lysate and medium in dsCaspase-1 treated group when compared to dsGFP (D).

Ca^2+^ has been reported to induce exocytosis in crayfish hemocytes [18] and regulates inflammasome activation and thus caspase-1 activation [17], [26]. Therefore, we investigated the effect of Ca^2+^ on proPO-casp release in *in vitro* experiments with isolated GCs. As shown in Figure 3A, the release of proPO and both proPO-casp fragments was Ca^2+^ and time dependent. When the antibody against the C-terminus of proPO was used, proPO but not active PO or proPO-casps could be detected. Again, this result suggests that the C-terminal fragments of proPO are rapidly degraded.

To confirm that the proPO-casp fragments are the result of a caspase-1-like cleavage, the effect of the caspase-1 inhibitors Z-YVAD-FMK or Ac-WEHD-FMK on the release of proPO-casp fragments was examined. The results presented in Figure 3B clearly show that the production of proPO-casps was markedly decreased in the presence of Z-YVAD (Figure 3B) or Z-WEHD-FMK (Figure S1). The amount of released proPO into the medium was also decreased in the presence of Z-YVAD-FMK, and a higher level of proPO in granular cell lysate was observed when the cells were incubated with caspase-1 inhibitors (Figure S1).

Furthermore, dsRNA caspase-1 treatment of granular hemocytes caused a complete reduction of the caspase-1 like transcript (Figure 3C), but no obvious reduction in protein level could be observed (data not shown). However, when the cells were treated with Ca^2+^ for 3 h to induce release of caspase-1 at 65 h of dsRNA treatment, the caspase-1 knockdown cells fail to produce new procaspase-1 like protein after another 24 h culture in L-15 (Figure 3D). The lower level of caspase-1 like protein resulted in a reduction of the levels of proPO-casp fragments both in cell lysate and medium. No change in total proPO protein could be observed after the RNAi treatment of granular cells (Figure 3D).

### Recombinant protein expression and purification

Because putative caspase-1 cleavage products were clearly detected outside GCs *in vitro* and *in vivo*, we decided to determine if these fragments possess biological functions. The N-terminal parts of proPO produced by cleavage at the putative caspase-1 cleavage site between Asp363 and Ala364 (proPO-casp1), and the fragment produced by cleavage between Asp389 and Asn390 (proPO-casp2) were produced as recombinant proteins with estimated sizes of 43 and 47 kDa, respectively. In addition, the N-terminal peptide fragment of proPO generated by cleavage by ppA between Arg176 and Thr177 [25] was produced (proPO-ppA).

**Figure 4:**
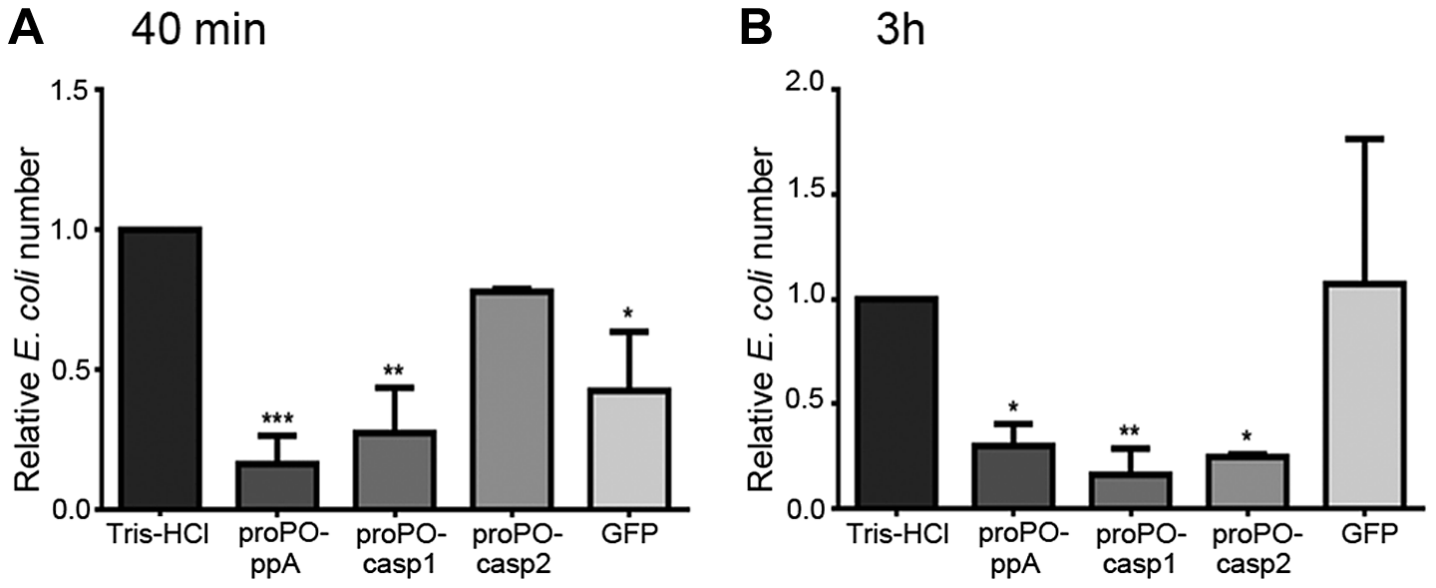
Bacterial clearance. The relative number of *E. coli* 40 min (A) and 3 h (B) after the injection of recombinant proPO fragments. The following were mixed with *E. coli* and injected separately: Tris-HCl (buffer control), proPO-ppA, proPO-casp1, proPO-casp2 and GFP (protein control). The relative number of *E. coli* in units of CFU/ml was calculated as the number for the specific fragments compared with the number for Tris-HCl. The results were analyzed using one-way ANOVA. * *P*<0.05, ** *P*<0.01, *** *P*<0.001 indicate a significant differences between the treatment and the Tris-HCl control. All experiments were repeated three times (N = 3). Each bar represents the mean ± SEM.

**Figure 5:**
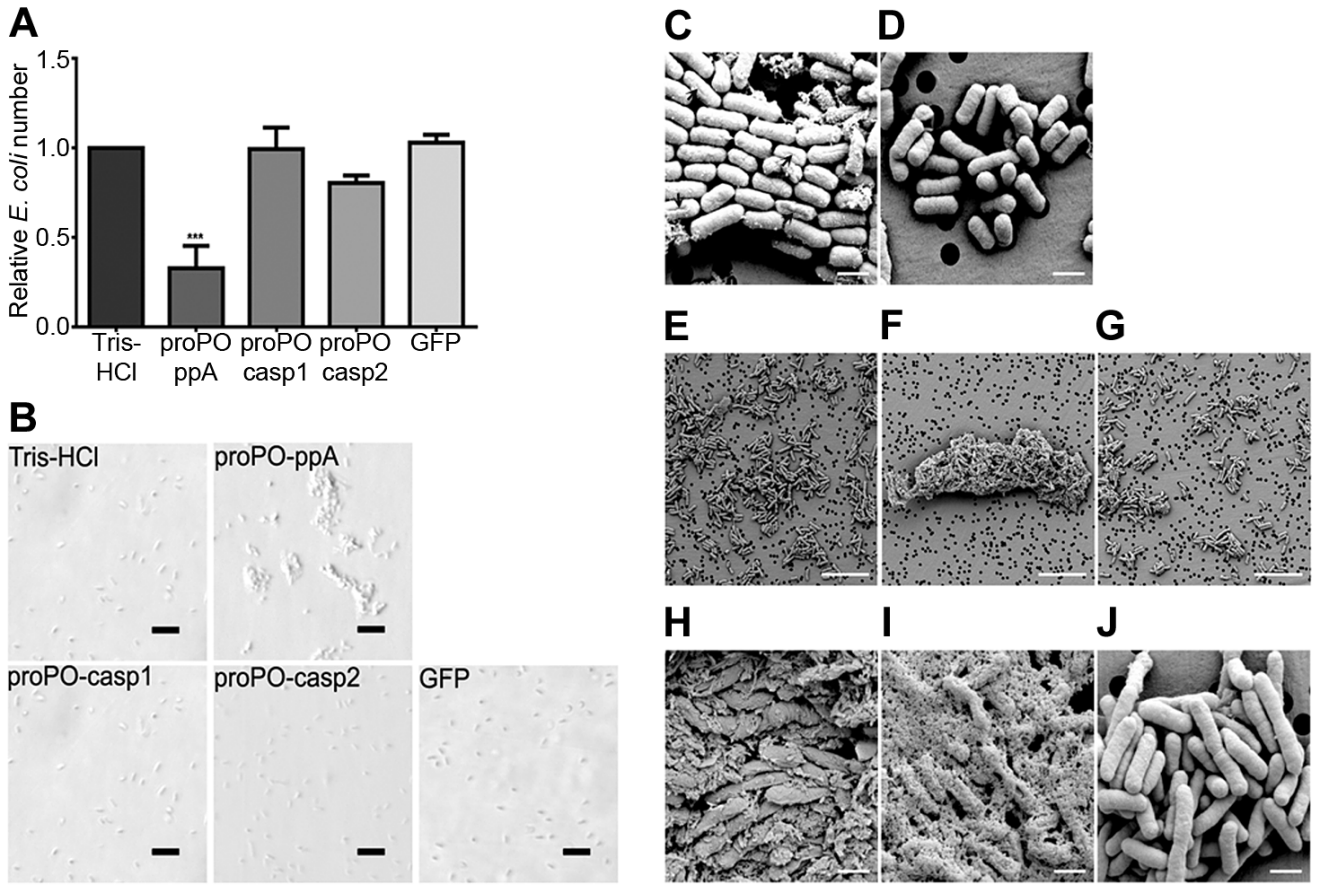
Antibacterial activity and agglutination. The number of *E. coli* after *in vitro* incubation with recombinant proPO fragments or GFP as a control, compared with a buffer control (A), was determined. All experiments were repeated at least three times (N = 3). Each bar represents the mean ± SEM, and *** *P*<0.001 indicates significant differences between the treatment and Tris-HCl. The data were analyzed by one-way ANOVA. *E. coli* observed by light microscopy after treatment with the recombinant fragments. The scale bars represent 10 µm (B). Changes in bacterial morphology after treatment with the proPO-ppA fragment as observed by SEM (C–J). After 15 min of incubation with proPO-ppA (C) or GFP (D), cell wall disruption started to appear and is reflected by longitudinal lines (black arrows in C) in the proPO-ppA treatment. The scale bars represent 1 µm. After 40 min of incubation with Tris-HCl (E), proPO-ppA (F), and GFP (G), agglutination was clearly observed in the proPO-ppA samples. The scale bars represent 10 µm. The *E. coli* cells were clearly distorted after proPO-ppA treatment (H–I) in contrast to the GFP treatment (J). The pictures shown in (H) and (I) were taken from different areas. The scale bars represent 1 µm.

To determine whether any of the proPO fragments are involved in the immune system, the bacterial clearance activities of these proteins were assessed. The fragments were mixed with bacteria and then injected into crayfish. Bacterial number in hemolymph was examined at 40 min and 3 h post injection. After 40 min, the *E. coli* titer was already significantly decreased in the proPO-ppA, proPO-casp1, and GFP treatment groups, whereas the injection of proPO-casp2 had no significant effect on the number of *E. coli* (Figure 4A). Because GFP also caused reduction of bacterial number at this time point, this might be a general protein effect. However, at 3 h post injection, the numbers of *E. coli* in the proPO-ppA, proPO-casp1, and proPO-casp2 injection groups were significantly lower than that in the non-protein injection groups and the GFP group (Figure 4B).The antimicrobial activities of all three fragments were then tested *in vitro* to determine if the bacterial clearance was caused by the proteins themselves or if other components were involved in the clearance process. The titer of *E. coli* decreased significantly after proPO-ppA treatment compared with the non-protein treatment, whereas the other fragments had no significant effect and did not exhibit any antibacterial activity (Figure 5A). When the treated bacteria were observed under the microscope, very strong agglutination was detected after treatment with the proPO-ppA peptide, whereas no signs of agglutination occurred with the proPO-casp fragments or GFP (Figure 5B). The minimal agglutinating concentration for proPO-ppA was the lowest for *E. coli* and *Staphylococcus aureus*, and the proPO-ppA fragment appeared to have the ability to agglutinate all of the tested bacterial species (Table 1). When the bacteria were observed by SEM after 15 and 40 min of incubation, we could see that proPO-ppA disrupted the *E. coli* cell morphology, causing the cell walls to shrink. After 15 min of incubation, the *E. coli* treated with proPO-ppA started to show signs of cell wall disruption, and a longitudinal line was observed (Figure 5C), in contrast to the GFP-treated bacteria (Figure 5D). After 40 min of incubation, the *E. coli* treated with proPO-ppA clearly formed clumps (Figure 5F), and the cells were flat (Figures 5H and 5I).

**Table 1:** Minimum agglutinating concentration of the proPO-ppA.

Microorganism		Minimal agglutinating concentration (µg/ml)
Gram positive bacteria	*S. aureus*	3.1
	*B. subtilis* ATCC6633	50
	*M. luteus*	6
Gram- negative bacteria	*E. coli* D21	1.5
	*A. hydrophila* B1	12
	*P. aeruginosa* OT97	6

**Figure 6:**
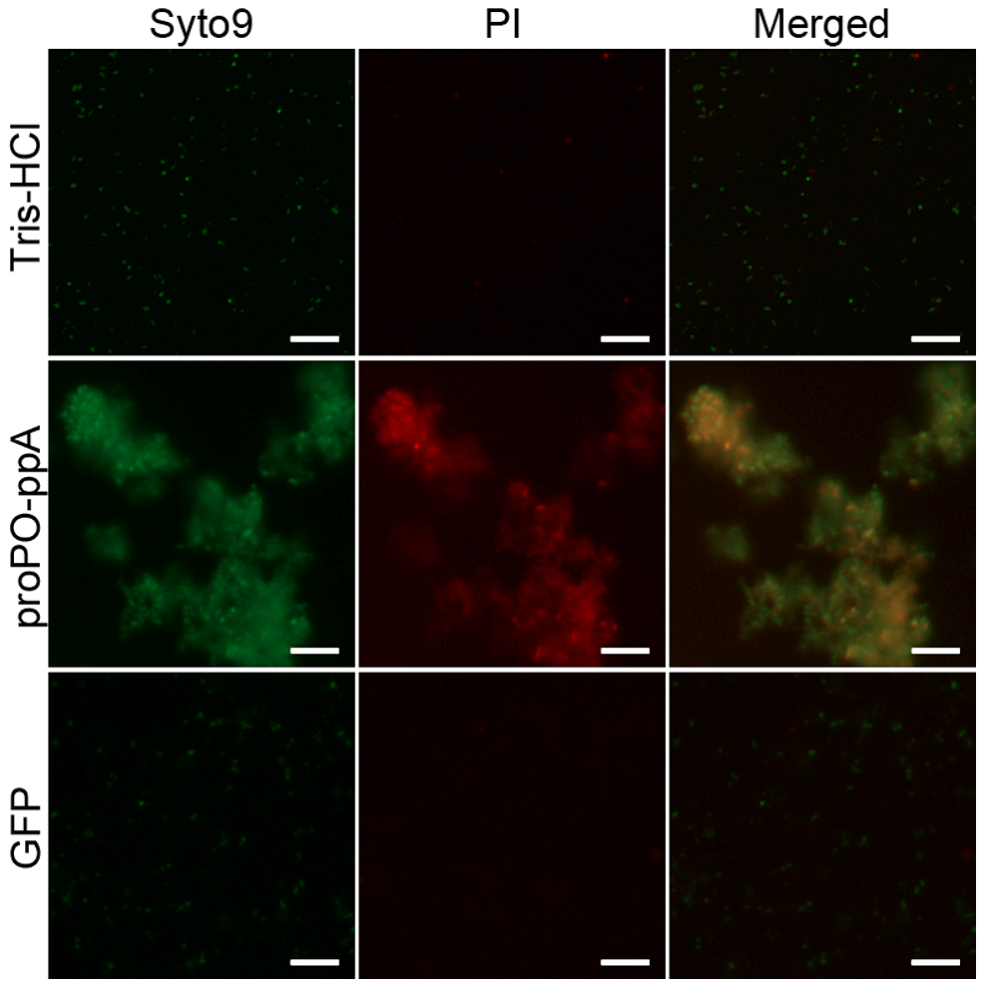
Bacterial viability. *E. coli* was incubated with recombinant proPO-ppA and stained with SYTO9 and PI to examine the viability of the bacteria. After staining, proPO-ppA-treated bacteria appeared to cluster, and a high proportion of the cells were stained red (dead cells), in contrast to the cells in the buffer and protein controls. The scale bars represent 10 µm.

Then, a bacteria viability assay was performed to determine if the strong agglutination killed the bacteria. Fluorescence microscopy clearly revealed that the proPO-ppA fragment greatly decreased the cell viability compared with the control treatments as measured by the red staining of dead bacteria (Figure 6). A few agglutinated bacteria were stained with only SYTO9 and appeared green in the merged picture (live cells).

## Discussion

The proPO system is an important innate immune response and is composed of a cascade of proteinases that terminates with the activation of the proenzyme proPO. After proteolytic cleavage, proPO becomes an active redox enzyme, PO, which forms melanin and other antimicrobial products in the non-catalytic pathway from quinone to melanin [1], [27], [28]. Because the product of PO is highly toxic, it is necessary to keep the proPO system under strict control to avoid deleterious effects of an activated proPO system, principally the redox enzyme PO. Several factors that can control this system have been described, including a multitude of proteinase inhibitors [1], [28], [29]. Moreover, if PO is generated, the melanization inhibition protein (MIPs) can inhibit melanin formation [4], [5]. Another way to protect against the inappropriate activation of proPO is to keep this proenzyme and its activation cascade in separate subcellular compartments. Thus, all arthropod proPOs are produced as leaderless proteins and are presumably located in the cytoplasm, whereas the activation system (proPO-AS) is located in secretory granules in crustaceans. This arrangement is similar to that of IL-1β, which is formed as a precursor in the cytoplasm and is then released to the outside of the cell during or after activation. Because caspase-1 cleavage is necessary for this activation and release steps and because such cleavage has been shown to be of importance for the secretion of several leaderless proteins [5], we looked for caspase-1 cleavage sites in proPO. We identified a new important regulator of the proPO system, caspase-1-like activity, which can efficiently cleave proPO at two cleavage sites and make the enzyme catalytically inactive. Moreover, we found that the N-terminal products of this cleavage have effects on bacterial clearance. There are no previous reports of inflammasomes in invertebrates, and NOD-like receptors (NLRs), which are part of the vertebrate caspase-1-activating inflammasome complex, have not been found in invertebrate genomes except that of the sea urchin [17]. However, both vertebrates and invertebrates express several pattern recognition receptors, and there might be other still undiscovered inflammasome sensor molecules responsible for the activation of invertebrate caspase-1-like activity. Recently, the structure of a *Drosophila* apoptosome composed of the Apaf-1-like protein Dark was reported. After binding to Dark, the initiator caspase Dronc cleaves the caspase DrICE and initiates an intrinsic cell death pathway [30]. The NLR-inflammasome and different apoptosomes are all examples of the oligomerization of CARD domain proteins involved in caspase activation, and their roles in cell death and immune responses are only beginning to be understood. Our discovery may add the proPO system to the list of immune responses balanced by such caspase regulation.

The activation of proPO by ppA occurs via proteolytic cleavage near the N-terminal, and a peptide of approximately 20 kDa is released during this process. Once activated, the PO activity must be strictly localized to where melanization is needed. In analogy with the complement system in vertebrates, we asked whether the cleaved activation peptide also has biological activities, as C3-cleaved peptides do.

To study whether this peptide (proPO-ppA) and the fragments resulting from caspase cleavage are involved in bacterial clearance, we injected *E. coli* together with these different fragments separately into crayfish and measured the number of bacteria in the hemolymph after the injection. Interestingly, all three peptides had the ability to decrease the bacterial number in the hemolymph compared to the injection of a control protein. To further investigate the mechanisms of action of these three peptides, we incubated each peptide directly with *E. coli* to identify any putative antibacterial activity, and we observed clear antibacterial activity for the proPO-ppA peptide. The observation of antibacterial activity in vitro suggests that the observed antimicrobial activity was a result of this peptide itself, whereas the proPO-casp1 and proPO-casp2 seem to require other components in crayfish to promote bacterial clearance. After incubation with the proPO-ppA fragment, both Gram-negative and Gram-positive bacteria were found to be heavily agglutinated. Moreover, live/dead staining of proPO-ppA-incubated *E. coli* revealed that the agglutinated bacteria contained a high percentage of dead cells. The generation of a highly antibacterial peptide after cleavage is similar to the case of hemocyanin, for which proteolytic cleavage in the crayfish plasma produces astacidin 1 [31]. These findings correspond to the antimicrobial function of human eosinophil cationic protein (ECP). Incubation with ECP can cause bacterial agglutination and decreased viability. One further example is the C-terminal region of human extracellular superoxide dismutase (SOD), which also exhibits antimicrobial activity against Gram-negative and Gram-positive bacteria [32], [33].

The SEM study showed that the antibacterial effect of proPO-ppA seemed to be on the bacterial cell wall. The cell wall appeared to shrink in the presence of proPO-ppA and then the *E. coli* cells were flattened. This antimicrobial activity is similar to the activity of funnel web spider venom on *Shigella sp.* [34].

**Figure 7:**
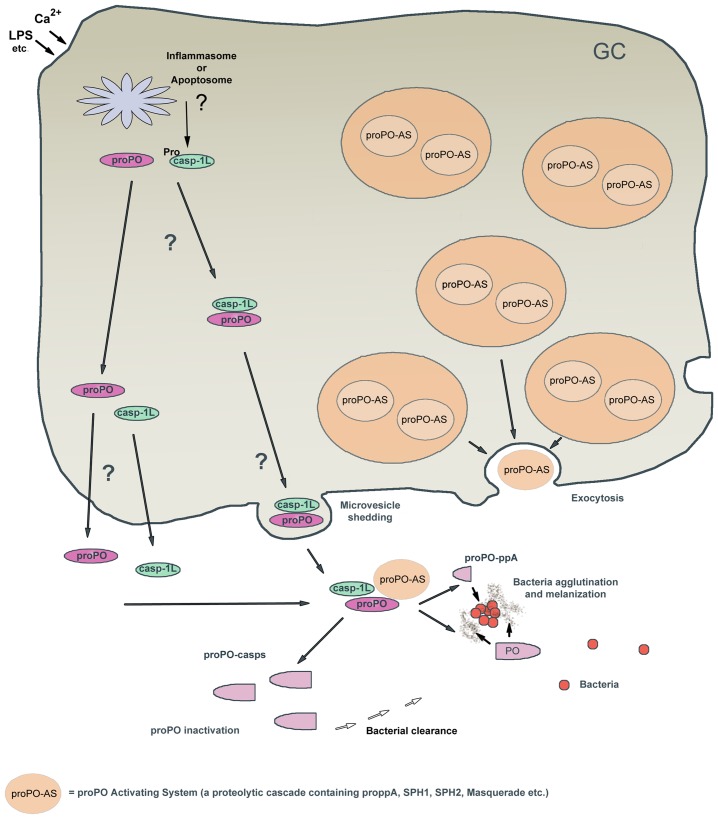
Hypothetical model of caspase-1 regulation of the proPO-AS. The proPO-AS is known to be localized to granules of the hemocytes and released by exocytosis after activation by microbial polysaccharides, while the final component proPO lacks a signal peptide and is located to the cytoplasm. In this paper we show that a caspase-1 like enzyme is involved in a Ca2+ -dependent release of proPO from the hemocytes and if proPO is not cleaved by the ppA then Caspase1-like enzyme will cleave proPO into two fragments that exhibit bacterial clearance activity and at the same time inactivate the melanin producing enzyme PO. Moreover we show that the peptide that is produced upon activation of proPO to PO by ppA possess high antibacterial and agglutinating activity. In summary this figure shows a putative mechanism for how the ProPO-system is localized to specific sites and is rapidly inactivated to prevent the spread of this dangerous enzyme. GC = granular cell.

We present new findings that may explain how the proPO system is regulated and how its activity is localized (Figure 7). After ppA cleavage, the small N-terminal proPO-ppA peptide causes the agglutination of bacteria at the site of infection, and PO activity then may localize melanization to these bacterial aggregates. We also provide evidence that the release of proPO, a leaderless protein, from cells may involve caspase-1-like activity, similar to that regulating IL-1βrelease. Furthermore, caspase-1-like cleavage of proPO inactivates the enzyme and generates two N-terminal fragments with bacterial clearing activity. These findings show that proPO is a multifunctional protein, with a phenoloxidase in the C-terminal region and an agglutinating and antimicrobial peptide in the N-terminal region, as well as N-terminal proPO-casps peptides with distinct biological activities.

## Materials and Methods

### Animals

Freshwater crayfish (*P. leniusculus*) was purchased and reared in a closed system at 10°C. Only healthy animals were used for the experiments.

### Antibodies

The antibody against human procaspase-1 and p20 subunit was purchased from Invitrogen. The antibody against kazal protease inhibitor (KPI) was from Santa Cruz Biotechnology (sc-46652). The ECL peroxidase-linked donkey anti-rabbit IgG (species-specific whole Ab) was purchased from GE Healthcare. The peroxidase-linked anti-goat IgG antibody (whole molecule) was purchased from Sigma. The FITC-conjugated goat anti-rabbit IgG (whole molecule, affinity isolated antigen-specific antibody) was purchased from Sigma.

### ProPO antibodies

To produce antibodies against the N-terminal and C-terminal peptides of proPO, proPO-N 1–76 and proPO-C 621–694 were cloned into the bacterial expression vector pGEX-4T-1 (GE Healthcare). Then, these plasmids were subsequently transformed into *Escherichia coli* cells (BL21), and a single colony was grown in LB medium containing 100 µg/ml ampicillin to OD_600_ = 0.5 and induced with 0.2 mM isopropyl β-d-thiogalactoside (IPTG) for 6 h at 20°C. Recombinant GST-fusion proteins were purified using GSTrap FF columns (GE Healthcare), and the GST tag was removed on the column by incubation with thrombin (GE Healthcare) at 4°C overnight. Then, the free recombinant peptides were eluted with PBS from the column. Two milligrams of recombinant proPO-N or proPO-C was used for the production of rabbit antiserum. The anti-proPO-N and anti-proPO-C antibodies were purified from the rabbit antiserum using GammaBind G-Sepharose (GE Healthcare) following the manufacturer's instructions.

### Immunostaining for proPO in granular cells (GCs)

GCs were separated using a 70% Percoll gradient in 0.15 M NaCl. The separated cells were resuspended in 0.15 M NaCl, seeded on coverslips, fixed and treated as described previously [35]. The immunostaining was performed using an antibody against the N-terminus of proPO (5 ng/µl, 1∶160) and a FITC-conjugated anti-rabbit antibody (1∶300). In addition, an antibody against a crayfish kazal protease inhibitor (KPI) was also used to counter stain hemocyte granules. The slides were mounted with VECTASHIELD mounting medium containing DAPI (Vector Laboratories). The stained cells were then observed under a fluorescence microscope.

### Hemocyte lysate and protein sample preparation

The hemolymph was centrifuged at 1000× g for 5 min at 4°C, and the hemocyte pellet was collected and washed two times with PBS. Then, hemocytes were lysed in PBS containing 2% Triton X-100 [15] and 1× protease inhibitor cocktail (Complete, Mini, EDTA-free, Roche). The cell lysate was centrifuged at 15,000× g for 15 min at 4°C, and the supernatant was collected. The protein concentration was determined, and 20 µg of protein was mixed with Laemmli sample buffer (62.5 mM Tris-HCl, 2% SDS, 10% (v/v) glycerol, 0.1 M DTT, 0.01% bromophenol blue, pH 6.8).

### Detection of caspase-1-like protein in crayfish

The presence of caspase-1-like protein was first examined in crayfish hemocyte lysate (20 µg protein) by western blotting using an antibody against human caspase-1. The hemocyte lysate was prepared as described above. In addition, the amount of the caspase-1-like protein in crayfish plasma was also determined in bacterial injected crayfish since bacterial components have been reported to be activators of inflammasomes and caspase-1 [36], [37]. To perform this experiment, crayfish (N = 3 for each group) were injected with 0.15 M NaCl, non-virulent *E. coli* or highly virulent *A. hydrophila* B1 [38], and hemolymph was collected at 1 h after injection. Hemocytes were removed from the hemolymph by centrifugation at 1000× g for 5 min at 4°C. Then, 200 µg of plasma protein was loaded onto 12.5% SDS-PAGE gels and subjected to western blotting as previously described [35]. The presence of caspase was detected using a rabbit anti-caspase antibody (1∶3000) and ECL peroxidase-linked donkey anti-rabbit IgG (GE Healthcare) (1∶7500). The detection of actin was also performed as a loading control using a goat anti-actin antibody (1∶5000) and an anti-goat secondary antibody (1∶5000).

### Caspase-1 activity assay

To determine caspase-1 activity, Ac-YVAD-pNA (Santa Cruz), a synthetic peptide and substrate for caspase-1, was used. Hemolymph was collected at 1 h after injection of 0.15 M NaCl, *E. coli* or *A. hydrophila* B1. Cell-free plasma samples were prepared as described above. Then 50 µl of plasma was mixed with 200 nM of Ac-YVAN-pNA in the presence or absence of the caspase-1 inhibitor, Z-YVAD-FMK (50 µM). The mixtures were incubated at 37°C for 1.5 h, and the absorbance was determined at 405 nM. The plasma without substrate was used as a negative control for each sample. The caspase-1 activity was reported as OD405/g plasma protein.

### Caspase-1 cleavage site prediction

To determine if there are any potential caspase-1 cleavage sites in *P. leniusculus* proPO, the amino acid sequence of proPO (GenBank: CAA58471) was analyzed using the bioinformatics tool PeptideCutter (http://web.expasy.org/peptide_cutter/).

### PO activity assay and detection of proPO-casp fragments in crayfish plasma

*E. coli* and *A. hydrophila* B1 were used to induce PO activity *in vivo*. Bacteria (1–3×10^7 ^CFU/100 µl) suspended in 0.15 M NaCl were injected into the crayfish (N = 5). The hemolymph was then collected before and 1 and 3 h after injection and centrifuged at 1000× g for 5 min to remove the hemocytes. Then, 30 µl of the cell-free plasma was used in a PO activity assay. The plasma was incubated for 30 min at room temperature (RT) with 20 µl of 3 mg/ml L-DOPA and 50 µl phosphate-buffered saline (PBS). The PO activity was determined by monitoring the absorbance at 490 nm, and a reaction mixture without substrate served as the baseline.

Cell-free plasma (250 µl) was centrifuged at 110,000× g at 4°C for 1.5 h to remove hemocyanin and 180 µl of the supernatant was subjected to acetone precipitation. Then, 1.6 µg of protein from each sample was loaded onto an SDS-PAGE gel, and the proPO-casps were detected by western blotting as described above using antibodies against the N-terminus or C-terminus of proPO.

### Detection of proPO-casp release *in vitro*

Because proPO is highly expressed in GCs, separated GCs were used in these experiments. The GCs were resuspended in 0.15 M NaCl and seeded into 96-well plates. After attachment for 10 min, the cells were incubated at RT in 10 mM HEPES-0.2 M NaCl buffer (pH 6.8) containing different concentrations of CaCl_2_ (0, 1, or 10 mM). Then, 50 µl of buffer was collected from each well after 30 and 60 min and subjected to TCA precipitation. The protein pellets were dissolved in the same volume of Laemmli sample buffer and analyzed by western blotting using antibodies against the N-terminus (1∶3000) or C-terminus (1∶3000) of proPO as described above.

To investigate the effect of the caspase-1 inhibitors, Z-YVAD-FMK (Tocris Bioscience) and Ac-WEHD-FMK (Santa Cruz) on proPO-casp release, GCs were incubated with 75 µl of HEPES-NaCl containing the inhibitor (0, 1, 10, or 50 µM) for 30 min before the addition of 75 µl of HEPES-NaCl containing 2 mM CaCl_2_. Then, the buffer was collected at 30 and 60 min, and the samples were prepared and analyzed as described above.

### Knockdown of Caspase-1-like protein *in vitro*

To knockdown crayfish caspase-1, double-stranded RNA (dsRNA) was synthesized with MEGAScript Kit (Ambion) and the template was amplified using the following primers (T7 promoter sequence is in italic); for dsCaspase-1 5′-*TAATACGACTCACTATAGGG*ACCTGTGGACCGACCTAGTGC-3′ and 5′-*TAATACGACTCACTATAGGG*GTCGGGCCTTTAGTTGGACACC-3′, dsGFP: 5′-*TAATACGACTCACTATAGGG*CGACGTAAACGGCCACAAGT-3′ and 5′-*TAATACGACTCACTATAGGG*TTCTTGTACAGCTCGTCCATGC-3′. The purified dsRNAs (1 µg/well) were then added to granular cells and maintained in 25% L-15 medium in 0.15 M NaCl at 16°C for 65 h (50% of medium was changed once at 48 h). The cells were then treated with 10 mM Ca^2+^ for 3 h to induce caspase-1 release. Next the cells were washed with 25% L-15 medium four times and then maintained in the medium for another 24 h to allow the cells to produce new caspase-1. Later, the cells were treated with 10 mM Ca^2+^ again, and same volume of medium was collected from each well at 60 min after incubation. In addition the granular cell lysate was also prepared from the 60-min Ca^2+^ treated cells. The samples were subjected to SDS-PAGE and the proPO-casp fragments and caspase-1 like protein were detected by western blotting.

### Cloning and recombinant protein expression

ProPO-ppA was amplified from *P. leniusculus* hemocyte cDNA using the following primers: proPO32EcoRI-F, (5′ TTTTTTGAATTCCAGGTGACCCAGAAGTTGCTGAGGA 3′) and proPOppA-R, (5′ CGCCTCGAGCTACCTGTTCACTTCAACCTGCATGCTT 3′). The PCR product was visualized by agarose gel electrophoresis, extracted from the gel and purified before being ligated into the pET32a expression vector between the *EcoR*I and *Xho*I restriction sites. The protein was expressed in *E. coli* BL21(DE3)pLysS cells. After the IPTG induction, the proteins, which were expressed in inclusion bodies, were refolded and purified with Ni-affinity chromatography.

Further, proPO-casp1 and proPO-casp2 were amplified using proPO-F (5′ CATGCCATGGGCCATCATCATCATCATCATCAGGTGACCCAGAAGTTGCTGAGGA 3′) and proPO-casp-R1 (5′ CGCCTCGAGCTAATCTGCCTCAAACGCGTCTCCTAAG 3′) for proPO-casp1 and proPOcasp-R2 (5′ CGCCTCGAGCTAGTCGTGACAGAATGCCAGCAGCACA 3′) for proPO-casp2. Both PCR products were ligated into the pET28b expression vector between the *Nco*I and *Xho*I restriction sites and expressed in the *E. coli* expression system as described above. Bacterial cells were disrupted by sonication, and the recombinant proteins were purified with Ni-affinity chromatography and dialyzed against 20 mM Tris-HCl, pH 8.0, at 4°C.

### *In vivo* bacterial clearance

Bacterial and protein injections were performed as follows. Briefly, wild-type *E. coli* were harvested at the mid-log phase, washed six times with 150 mM NaCl at 1200×g for 5 min and resuspended in 150 mM NaCl at 1×10^9^ CFU/ml. Bacterial suspensions (100 µl) were mixed with 20 µg of recombinant protein or 20 mM Tris-HCl and injected into the crayfish at the base of a walking leg. At 40 min and 3 h after injection, hemolymph was collected, serially diluted with 150 mM NaCl and plated on LB agar. The plates were incubated at 37°C overnight. The number of bacterial colonies was counted, and the number of CFU per ml was calculated for each treatment.

### *In vitro* bacterial clearance

To investigate whether the bacterial clearance activity was a direct effect of the protein fragments, we performed an *in vitro* bacterial clearance assay as follows. *E. coli* were prepared as described above, and 100 µl of resuspended *E. coli* was mixed with 20 µg of recombinant protein. Then, the volume was adjusted to 1 ml with 150 mM NaCl. The mixtures were incubated for 1 hour at room temperature with mild agitation, serially diluted and plated onto LB agar to calculate the CFU per ml. The plates were observed under a microscope.

### Agglutination assay

The minimum protein concentration for bacterial agglutination was tested as previously described [39]. The bacteria used in the experiment were *Staphylococcus aureus* Cowan, *Micrococcus luteus* M III, *E. coli* D21, *A. hydrophila* B1, and *Pseudomonas aeruginosa* OT97. Overnight cultures of bacteria were collected and washed three times in 150 mM NaCl. Each bacterial species was resuspended, and the optical density was adjusted to 2. The recombinant proteins were twofold serially diluted, and 50 µl of each dilution was mixed with 50 µl of bacterial suspension and incubated at room temperature for 1 hour.

### Bacteria viability assay

*E. coli* and protein mixtures were prepared as described for the *in vitro* bacterial clearance assay. After 5 min of incubation, SYTO9 (Invitrogen) was added to a final concentration of 50 nM, and propidium iodide (PI) was added to a final concentration of 1 µg/ml. Then, the samples were visualized with a fluorescence microscope.

### SEM analysis

*E. coli* at O.D. 0.5 (100 µl) was incubated with 20 µg of the proPO-ppA peptide for 40 min at room temperature with mild agitation. After incubation, the bacteria were harvested and fixed with glutaraldehyde following standard procedures for SEM.

## Supporting Information

Figure S1 **Inhibitory effect of Ac-WEHD-FMK, a caspase-1 inhibitor.** Granular cells (GC) were incubated in buffer containing different concentrations of Ca^2+^. The presence of released proPO-casps was examined at 60 min. The release of proPO-casps was inhibited when the cells were preincubated for 30 min with the caspase-1 inhibitor Ac-WEHD-FMK prior to Ca^2+^ addition. In addition, the proPO level inside the cell was increased in the presence of the inhibitor.
(TIF)
