# Correction: Caspase-1-Like Regulation of the proPO-System and Role of ppA and Caspase-1-Like Cleaved Peptides from proPO in Innate Immunity

**DOI:** 10.1371/journal.ppat.1005665

**Published:** 2016-05-20

**Authors:** Miti Jearaphunt, Chadanat Noonin, Pikul Jiravanichpaisal, Seiko Nakamura, Anchalee Tassanakajon, Irene Söderhäll, Kenneth Söderhäll

The authors would like to correct Fig 5. In Fig 5B, the proPO-casp1 and Tris-HCL result was mistakenly duplicated, and in the corrected figure new correct panels are mounted for proPO-casp1 and Tris-HCl.

**Fig 5:**
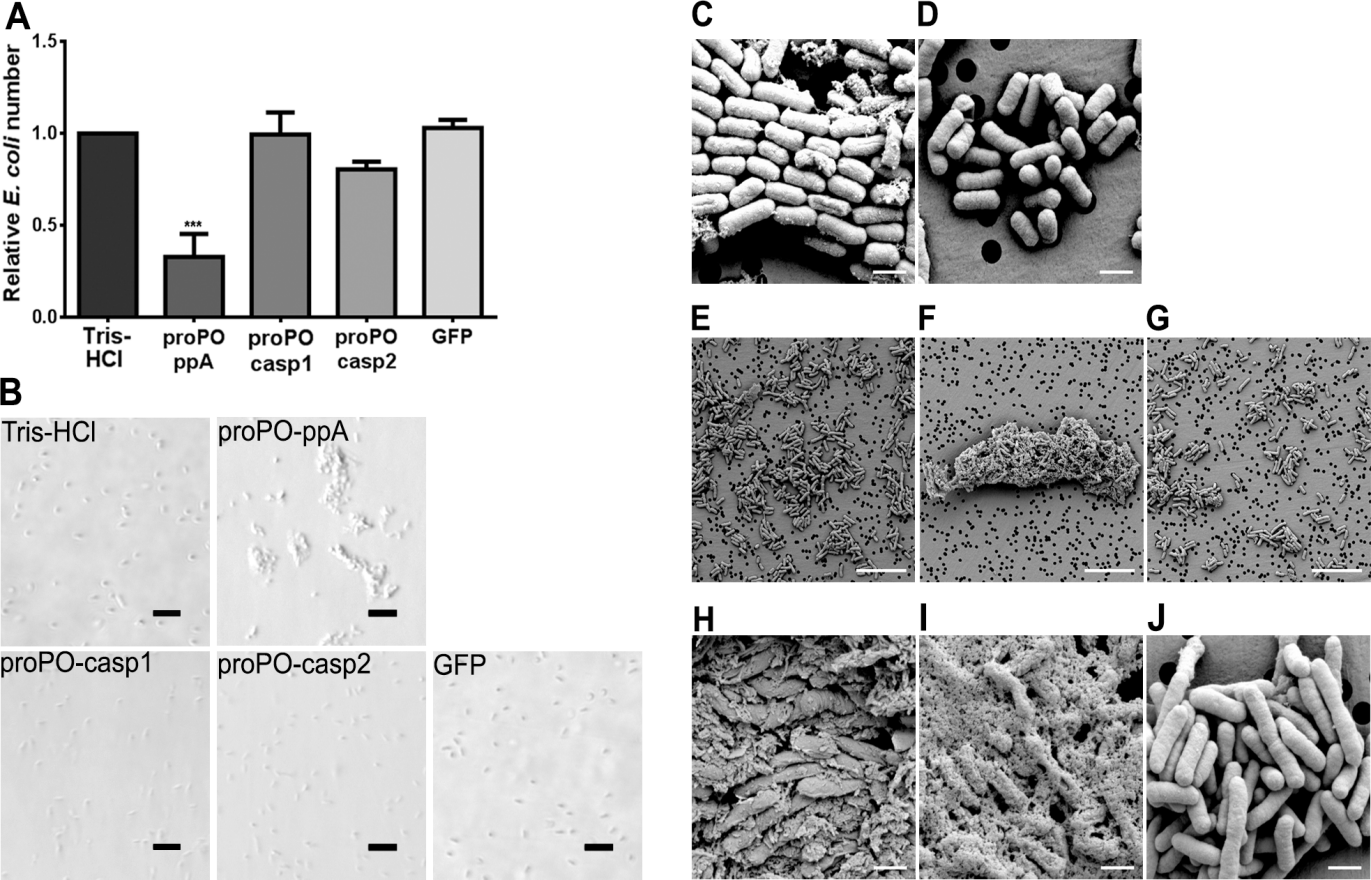
Antibacterial activity and agglutination. The number of *E*. *coli* after *in vitro* incubation with recombinant proPO fragments or GFP as a control, compared with a buffer control (A), was determined. All experiments were repeated at least three times (N = 3). Each bar represents the mean ± SEM, and *** *P*<0.001 indicates significant differences between the treatment and Tris-HCl. The data were analyzed by one-way ANOVA. *E*. *coli* observed by light microscopy after treatment with the recombinant fragments. The scale bars represent 10 μm (B). Changes in bacterial morphology after treatment with the proPO-ppA fragment as observed by SEM (C–J). After 15 min of incubation with proPO-ppA (C) or GFP (D), cell wall disruption started to appear and is reflected by longitudinal lines (black arrows in C) in the proPO-ppA treatment. The scale bars represent 1 μm. After 40 min of incubation with Tris-HCl (E), proPO-ppA (F), and GFP (G), agglutination was clearly observed in the proPO-ppA samples. The scale bars represent 10 μm. The *E*. *coli* cells were clearly distorted after proPO-ppA treatment (H–I) in contrast to the GFP treatment (J). The pictures shown in (H) and (I) were taken from different areas. The scale bars represent 1 μm.

The authors confirm that these changes do not alter their findings. The authors have provided raw, uncropped photos with indications of the cropped area for each treatment as Supporting Information.

## Supporting Information

S1 File Uncropped images. The uncropped images of *E*. *coli* agglutination after incubation with Tris-HCl, proPO-ppA fragment, proPO-casp1 fragment, proPO-casp2 fragment, and GFP as indicated. The cropped areas used for Fig 5B are indicated with red lines.
(ZIPX)
