# Supplementary figures and images for: Caspase-1-Like Regulation of the proPO-System and Role of ppA and Caspase-1-Like Cleaved Peptides from proPO in Innate Immunity
Source: PLoS Pathog. 2014 Apr 10;10(4):e1004059. doi: 10.1371/journal.ppat.1004059 (PMC3983073; doi:10.1371/journal.ppat.1004059)

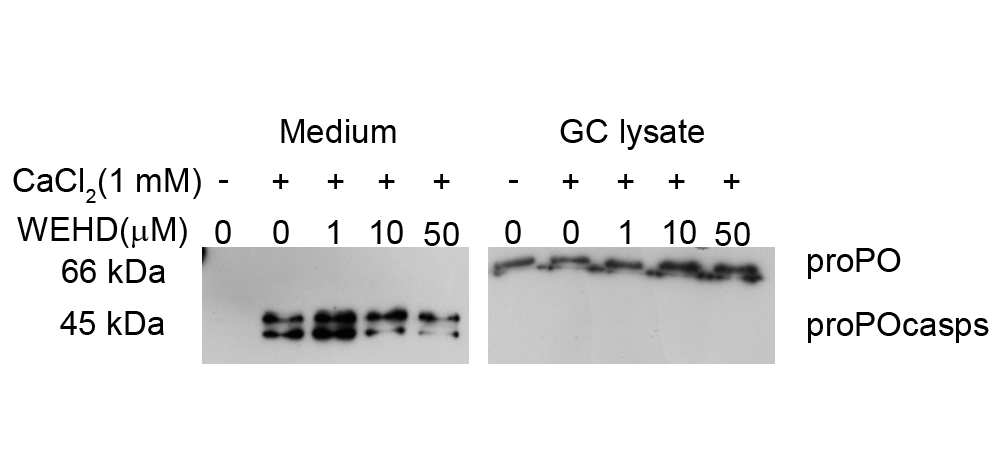

Supplement: Figure S1 — Inhibitory effect of Ac-WEHD-FMK, a caspase-1 inhibitor. Granular cells (GC) were incubated in buffer containing different concentrations of Ca2+. The presence of released proPO-casps was examined at 60 min. The release of proPO-casps was inhibited when the cells were preincubated for 30 min with the caspase-1 inhibitor Ac-WEHD-FMK prior to Ca2+ addition. In addition, the proPO level inside the cell was increased in the presence of the inhibitor. (TIF) [file ppat.1004059.s001.tif]
