# Supplementary figures and images for: Correction: Caspase-1-Like Regulation of the proPO-System and Role of ppA and Caspase-1-Like Cleaved Peptides from proPO in Innate Immunity
Source: PLoS Pathog. 2016 May 20;12(5):e1005665. doi: 10.1371/journal.ppat.1005665 (PMC4874688; doi:10.1371/journal.ppat.1005665)

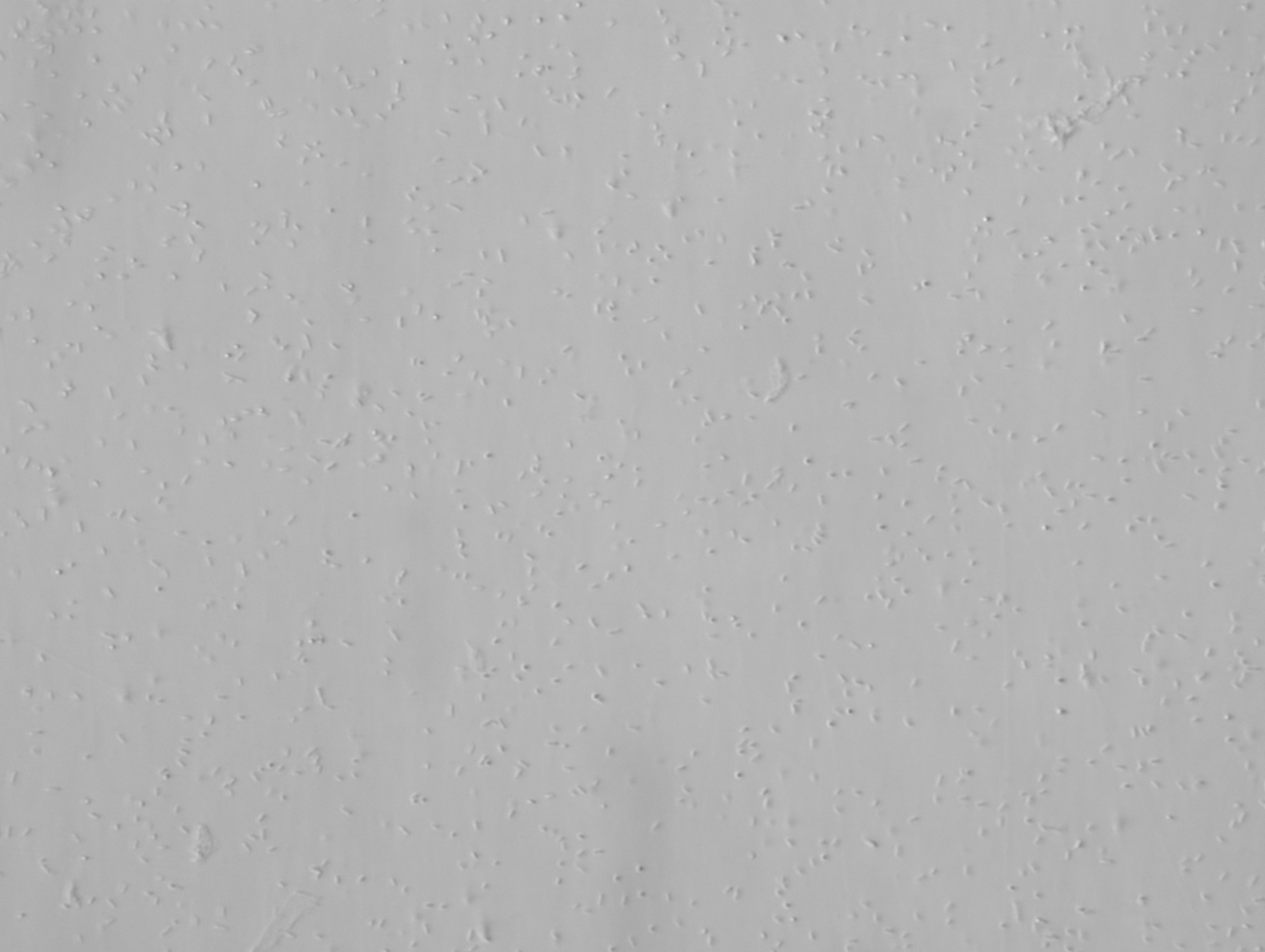

Supplement: S1 File — The uncropped images of E. coli agglutination after incubation with Tris-HCl, proPO-ppA fragment, proPO-casp1 fragment, proPO-casp2 fragment, and GFP as indicated. The cropped areas used for Fig 5B are indicated with red lines. (ZIPX) [file ppat.1005665.s001.zipx › 3_1C2130403_008.TIF]

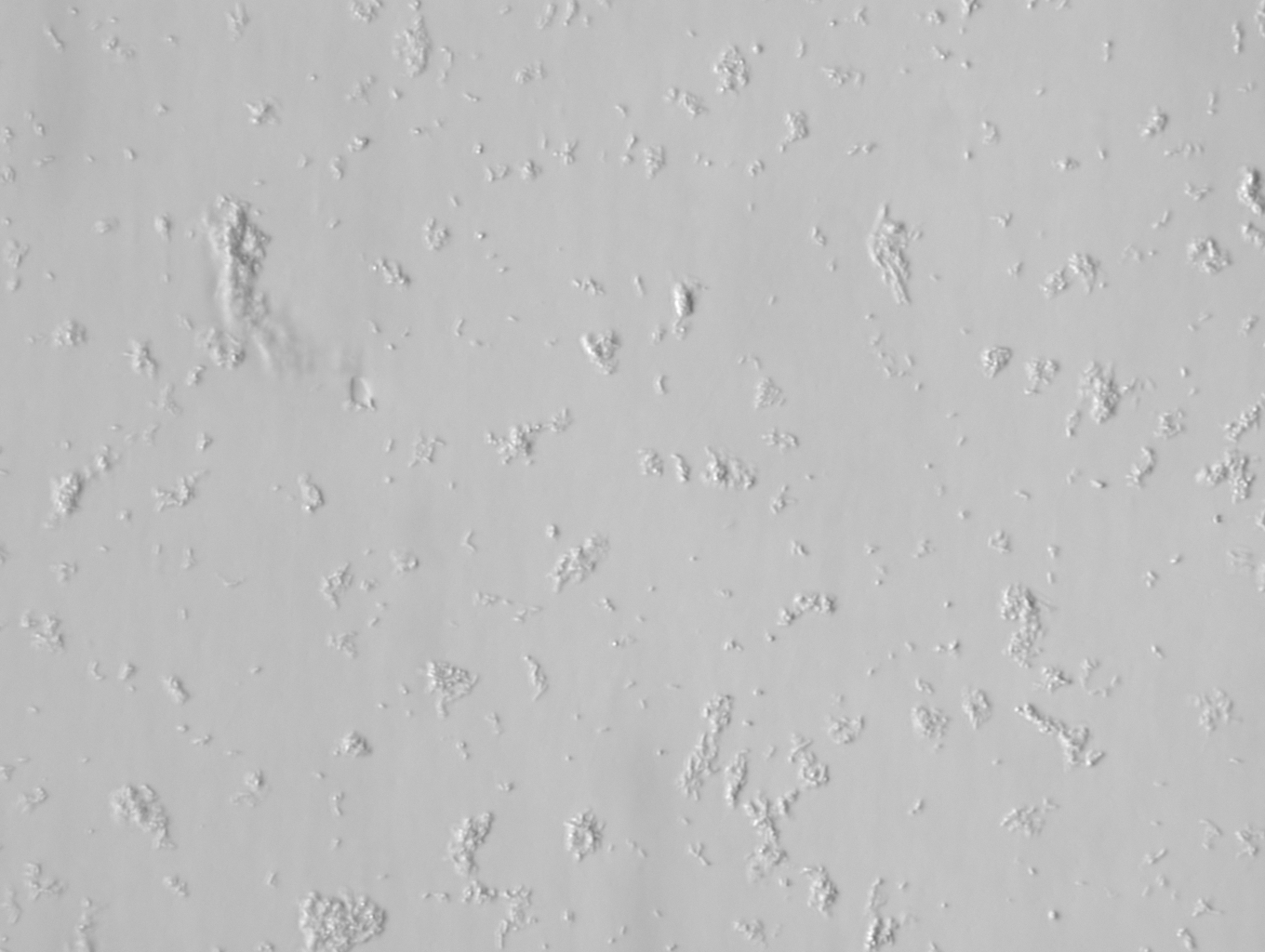

Supplement: S1 File — The uncropped images of E. coli agglutination after incubation with Tris-HCl, proPO-ppA fragment, proPO-casp1 fragment, proPO-casp2 fragment, and GFP as indicated. The cropped areas used for Fig 5B are indicated with red lines. (ZIPX) [file ppat.1005665.s001.zipx › 4_1POA130403_011.TIF]

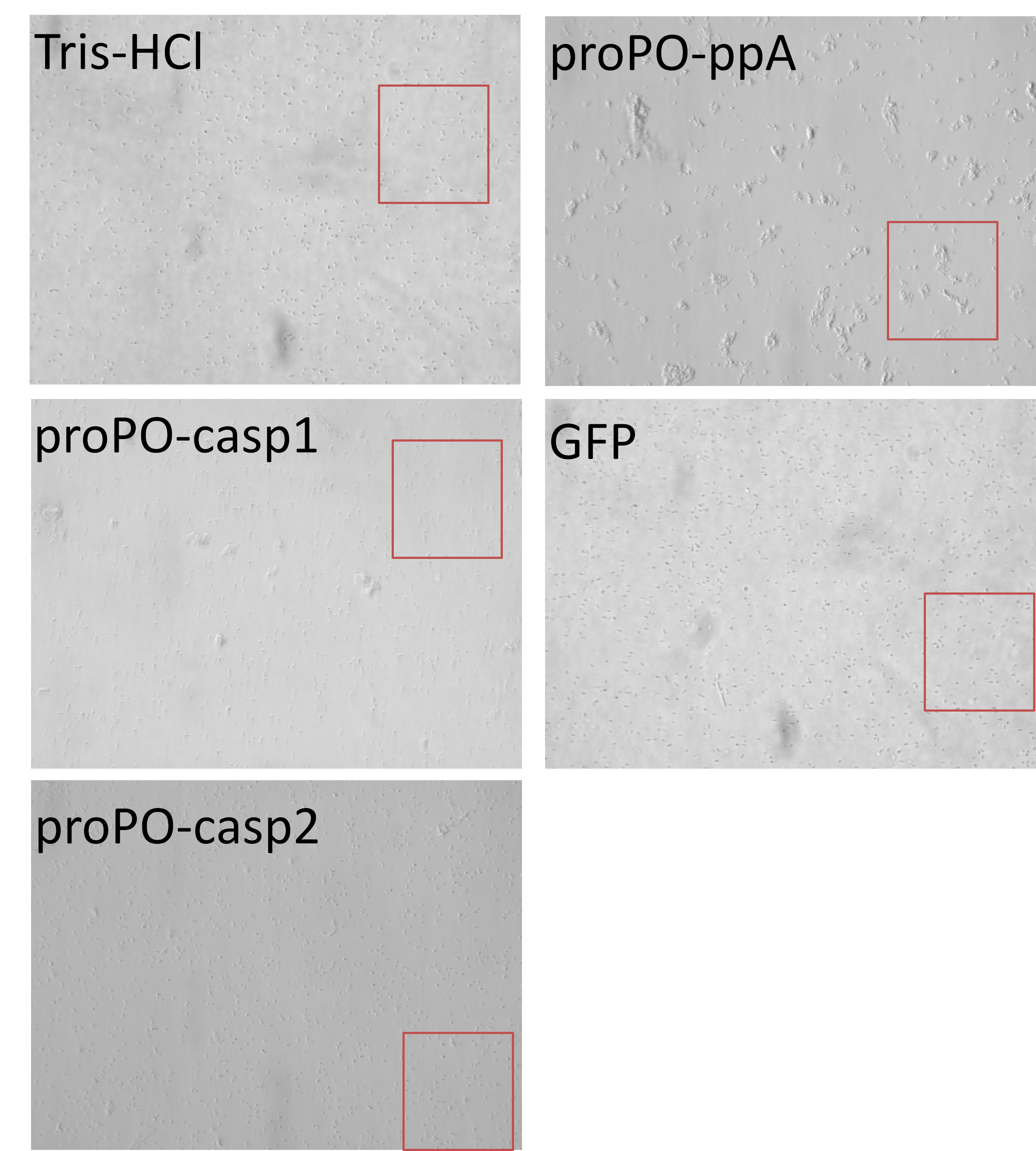

Supplement: S1 File — The uncropped images of E. coli agglutination after incubation with Tris-HCl, proPO-ppA fragment, proPO-casp1 fragment, proPO-casp2 fragment, and GFP as indicated. The cropped areas used for Fig 5B are indicated with red lines. (ZIPX) [file ppat.1005665.s001.zipx › Cropped locations.tif]

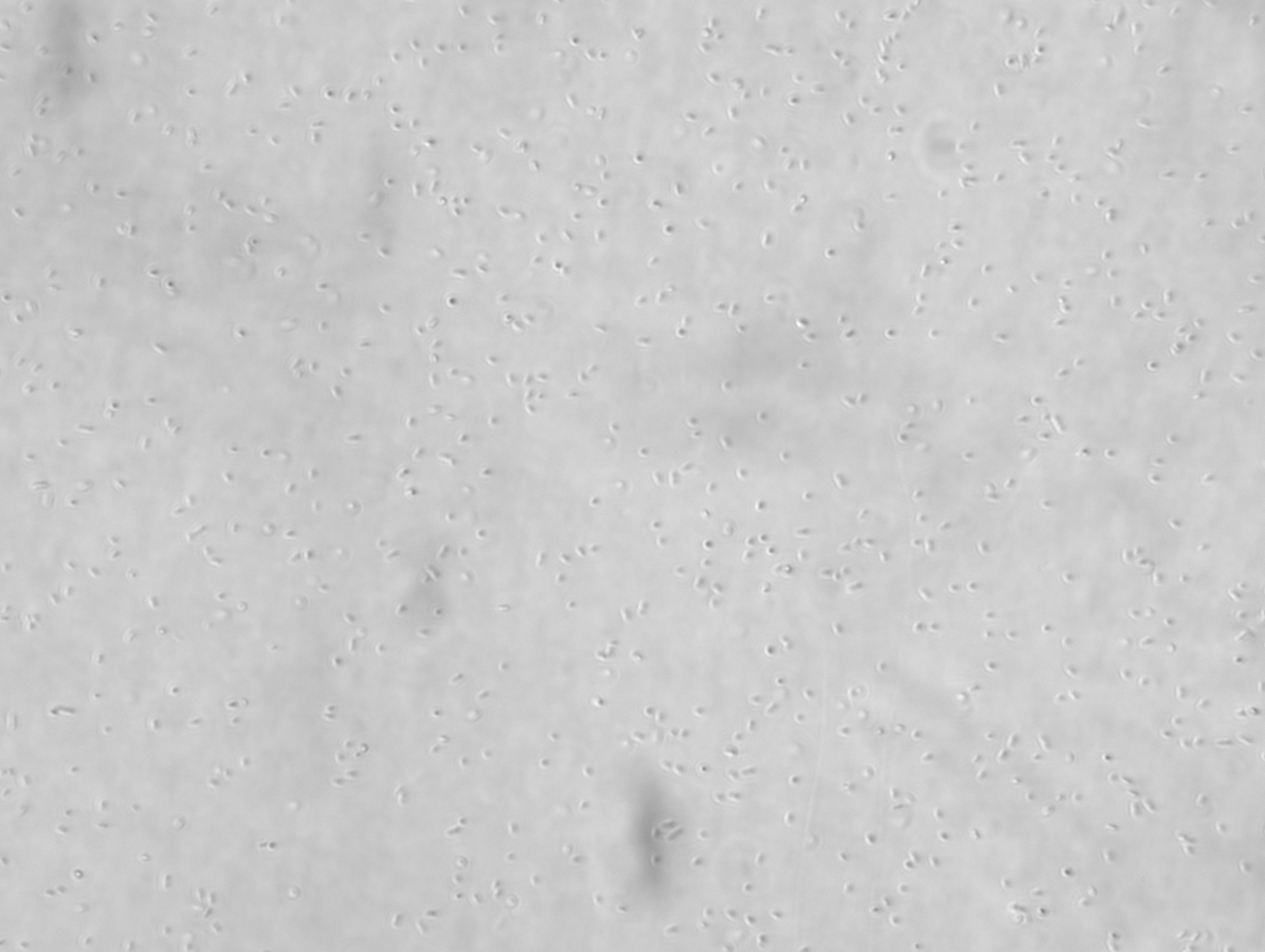

Supplement: S1 File — The uncropped images of E. coli agglutination after incubation with Tris-HCl, proPO-ppA fragment, proPO-casp1 fragment, proPO-casp2 fragment, and GFP as indicated. The cropped areas used for Fig 5B are indicated with red lines. (ZIPX) [file ppat.1005665.s001.zipx › 1_1Tris130403_002.TIF]

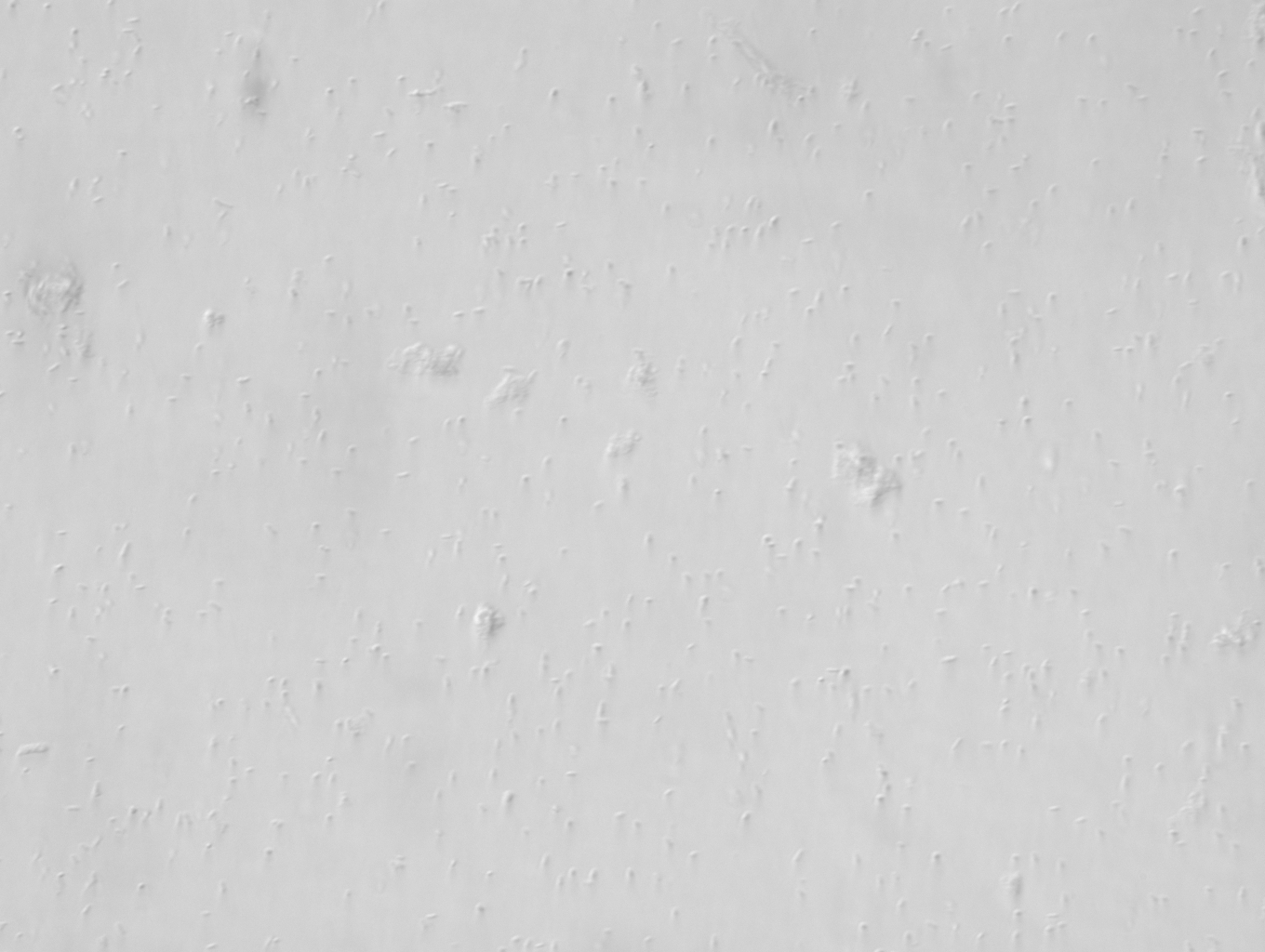

Supplement: S1 File — The uncropped images of E. coli agglutination after incubation with Tris-HCl, proPO-ppA fragment, proPO-casp1 fragment, proPO-casp2 fragment, and GFP as indicated. The cropped areas used for Fig 5B are indicated with red lines. (ZIPX) [file ppat.1005665.s001.zipx › 2_1C1 130403_005.TIF]

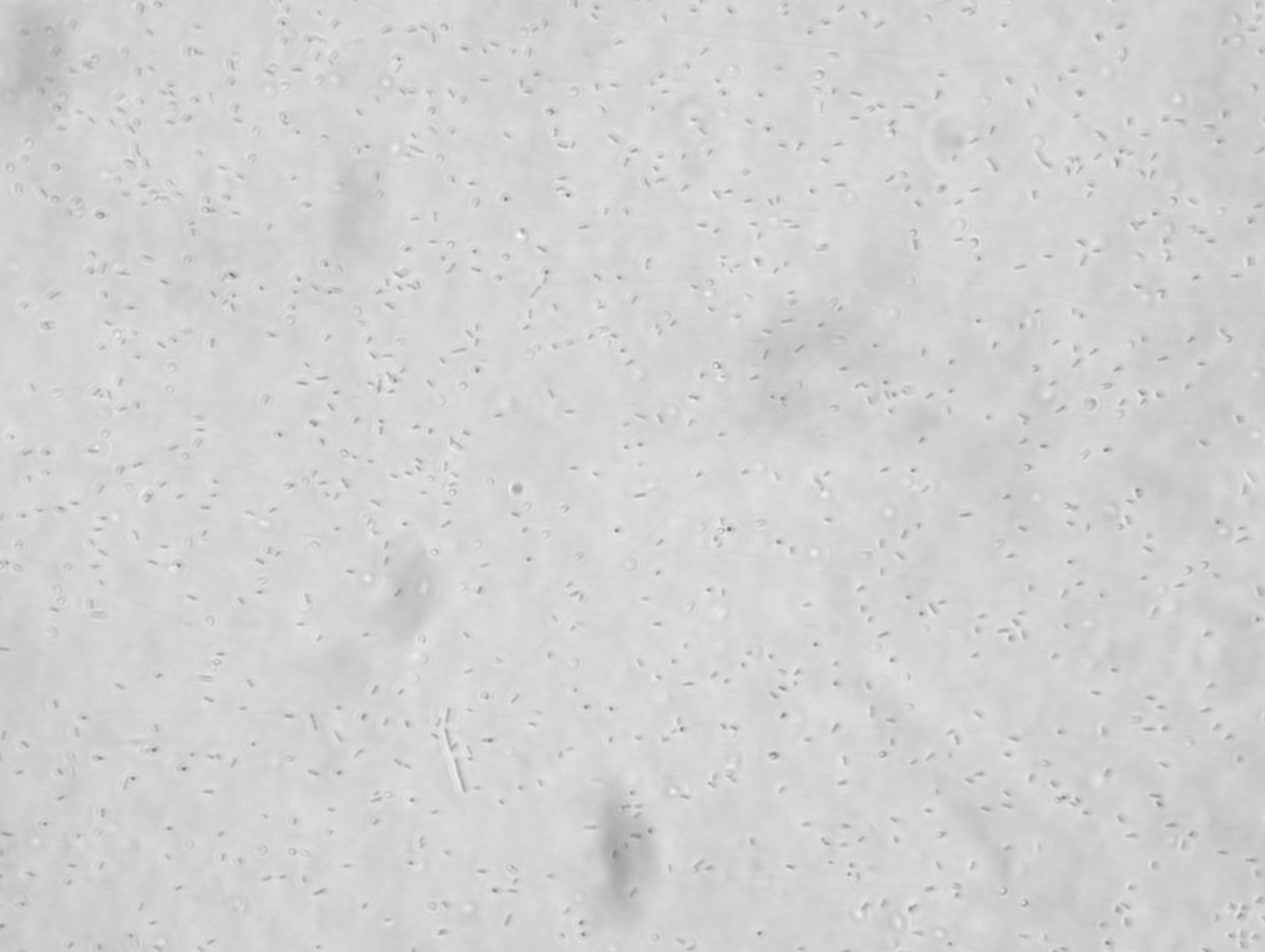

Supplement: S1 File — The uncropped images of E. coli agglutination after incubation with Tris-HCl, proPO-ppA fragment, proPO-casp1 fragment, proPO-casp2 fragment, and GFP as indicated. The cropped areas used for Fig 5B are indicated with red lines. (ZIPX) [file ppat.1005665.s001.zipx › 5_GFP130403_005.TIF]
